# Experimental Evolution of Reduced Susceptibility to Artemether–Lumefantrine in *Plasmodium berghei* Following Serial Drug Pressure

**DOI:** 10.1155/japr/1895485

**Published:** 2026-09-28

**Authors:** Stephen Mwangi Njoroge, Shadrack Kanyonji Kimani, Teresia Wambui Ngonjo, Maureen Stella Olunga, Julius Seng′ete Wambua, Milka Wambui Waithera, Hamida Yahaya Wawa, James Ng′ang′a Waweru

**Affiliations:** ^1^ Department of Biological and Physical Sciences, School of Pure and Applied Sciences, Karatina University, Karatina, Kenya, karu.ac.ke; ^2^ Department of Medical Laboratory Science, Medical School, Mount Kenya University, Thika, Kenya, mku.ac.ke; ^3^ Department of Biochemistry, School of Biomedical Sciences, Jomo Kenyatta University of Agriculture and Technology, Nairobi, Kenya, jkuat.ac.ke; ^4^ Department of Biochemistry and Molecular Biology, Muhimbili University of Health and Allied Sciences, Dar es Salaam, Tanzania, muchs.ac.tz; ^5^ Department of Physical and Biological Science, Murang′a University of Technology, Murang′a, Kenya

**Keywords:** artemether–lumefantrine resistance, drug selection pressure, in vivo, malaria, *Plasmodium berghei*

## Abstract

Malaria remains a major global health problem, with uneven progress and the greatest disease burden clustered in sub‐Saharan Africa. Antimalarial drug resistance continues to undermine malaria control efforts, with widespread resistance reported in *Plasmodium falciparum,* including reduced susceptibility to artemisinin‐based combination therapies (ACTs). However, despite increasing concerns over the long‐term effectiveness of ACTs, the processes through which resistance to artemether–lumefantrine (AL) partner drugs evolves and contributes to resistance against individual drug components remain poorly understood. Consequently, we investigated AL resistance in *Plasmodium berghei ANKA* towards understanding resistance development to full ACT regimens rather than individual drugs. Firstly, drug efficacy was evaluated using the standard 4‐day suppressive test (4‐DT), with baseline effective doses (ED_50_ and ED_90_) for the parental line determined as 1.45 and 2.80 mg/kg, respectively. Following 20 stepwise in vivo drug selection pressure passages, from the parental (0th) to the 20th passage in mice over 26 weeks, resistance increased by 2.85‐fold in ED_50_ and 2.65‐fold increase in ED_90_, indicating reduced drug susceptibility and stepwise resistance selection. After five drug‐free passages and recovery following 45 days of cryopreservation at −80°C, the parasite retained slight resistance across all assays, indicating a stable resistance phenotype. In conclusion, continuous drug exposure successfully led to the development of *P. berghei* parasites with reduced sensitivity to AL, as evidenced by significantly lower chemo‐suppression in resistant lines compared with sensitive controls (*p* < 0.05). These findings demonstrate that AL resistance can be progressively selected over a period of time and stability maintained in *P. berghei*, providing insights into the durability and evolutionary vulnerability of ACTs under sustained drug pressure. Future studies should enhance the level of resistance, evaluate its stability, and investigate the molecular basis of AL resistance to identify reliable genetic markers for monitoring resistance to ACTs.

## 1. Introduction

Malaria remains one of the key parasitic diseases globally [1], with an estimated 282 million cases and 610,000 deaths reported in 2024, most of which occurred in sub‐Saharan Africa and among children under 5 years of age [2]. *Plasmodium falciparum* and *Plasmodium vivax* are the major causative agents of malaria worldwide [3], although *P. falciparum* is responsible for most severe disease and mortality in Africa. Although WHO‐approved malaria vaccines such as RTS,S/AS01 and R21/Matrix‐M, together with emerging candidates including PfSPZ, RH5, and transmission‐blocking vaccines, are expanding malaria prevention strategies; chemotherapy remains the primary approach for malaria treatment and control [4].

Several Artemisinin‐based combination therapies (ACTs) are currently the recommended first‐line treatment for human malaria [5]. Specifically, artemether–lumefantrine (AL) remains a cornerstone in the treatment of uncomplicated malaria due to its high efficacy, rapid parasite clearance, and widespread use in malaria‐endemic regions [6]. Combined with vector control interventions, ACTs have significantly contributed to reducing malaria burden globally [7]. However, the continued effectiveness of ACTs is threatened by the emergence and spread of antimalarial drug resistance, particularly reduced susceptibility to artemisinin derivatives and their partner drugs [8].

AL is among the most widely used ACT regimens because it combines two drugs with different pharmacological properties and mechanisms of action [9]. Artemether (ART) is a short‐acting artemisinin derivative that rapidly kills parasites through heme‐dependent activation and generation of reactive oxygen species, particularly during the early ring stages of parasite development [10]. In contrast, lumefantrine (LM) is a long‐acting partner drug that inhibits heme detoxification in the parasite digestive vacuole and maintains prolonged antimalarial activity due to its extended half‐life [11]. This pharmacokinetic and mechanistic complementarity reduces the likelihood of parasite survival and delays resistance emergence compared with monotherapies. Nevertheless, the short half‐life of ART and prolonged persistence of LM may create a temporal window of subtherapeutic exposure, especially in high‐transmission settings, allowing partially tolerant parasites to survive and gradually adapt under continuous drug pressure [12]. Reduced susceptibility to artemisinin and their derivatives does not necessarily lead to treatment failure, as the partner drug can still eliminate the infection. Nevertheless, emerging concerns have been reported. Therapeutic efficacy studies evaluating AL have documented treatment failure rates exceeding 10% [13]. These observations, reported in Burkina Faso [14], Angola [15]), and Uganda [16] underscore the need for continued surveillance, although definitive evidence of evolution of combination of AL resistance remains lacking. Therefore, we sought to investigate the experimental evolution of AL resistance in *Plasmodium berghei* under sustained drug pressure, with the aim to understand the dynamics, stability, and early evolution of ACT resistance.

Experimental studies using *P. berghei* models have demonstrated that resistance to antimalarial drugs can be induced progressively through serial drug exposure. For example, successful induction of LM‐ and PQ‐resistant [17] and PRD‐resistant [18–20] parasite lines was previously described. Similarly, a recent study demonstrated that ART resistance can be achieved in vivo [21]. These studies consistently showed that resistance develops gradually rather than abruptly, supporting the concept of stepwise adaptation under continuous pharmacological selection. Interestingly, monotherapy‐associated resistance was reported to develop more rapidly than resistance associated with LM, likely due to stronger single‐drug selection pressure. In contrast, ACTs generally slow resistance evolution because parasites must simultaneously tolerate drugs with different modes of action and pharmacokinetic profiles.

Further studies have shown that resistance evolution is influenced not only by drug pressure but also by biological fitness costs and resistance stability. Pinheiro et al. [22] reported that LM‐ and PQ‐resistant *P. berghei* lines exhibit different stability profiles under drug‐free conditions, with piperaquine resistance remaining relatively stable, whereas LM‐associated resistance appeared partially transient. Similarly, measurable fitness trade‐offs have been reported in ART‐resistant parasites [21]. Besides, evidence from Cambodia demonstrated that delayed parasite clearance by artemisinin derivatives increased dependence on individual partner drugs, accelerating the emergence of combination therapy failure. Similar trends are developing in sub‐Saharan Africa, raising concerns about the long‐term durability of current antimalarial regimens [23–25]. Consequently, investigating the experimental evolution of AL resistance in *P. berghei* provides a valuable model for understanding resistance development, identifying genetic markers for monitoring resistance and prediction of the future effectiveness of ACT therapy.

To date, experimental resistance‐selection studies in *P. berghei* have primarily focused on individual antimalarial agents generating parasite lines with reduced susceptibility or resistance to ART, LM, piperaquine, and pyronaridine [17–21]. These studies have substantially advanced understanding of single‐drug resistance mechanisms; however, no study has experimentally selected parasites under the combined pressure of AL, despite the drug being coadministered clinically as first‐line therapy across malaria‐endemic countries sub‐Saharan Africa. Resistance evolving under combination drug pressure may follow distinct evolutionary mechanisms compared with resistance selected by either partner drug alone because parasites are exposed to simultaneous selective pressures. Consequently, adaptive mechanisms selected under AL treatment cannot be reliably inferred from studies of ART or LM monotherapy. The present study addresses this knowledge gap by providing the first experimental characterization of the stepwise development of reduced AL susceptibility in *P. berghei*, susceptibility profile and phenotypic stability under sustained AL exposure. This work establishes a clinically relevant experimental model for investigating the early evolutionary processes that may precede ACT resistance.

## 2. Materials and Methods

### 2.1. Experimental Animals and Parasites

Experimental mice consisted of randomly bred male Swiss albino mice aged 6–7 weeks and weighing 18–22 g. The mice were housed in well‐labeled standard Macrolon Type II cages under controlled environmental conditions of 22°C and 60%–70% relative humidity, with free access to commercial rodent feed and water ad libitum throughout the study.

A transgenic *P. berghei ANKA* strain expressing green fluorescent protein (GFP) was used for resistance selection studies. Cryopreserved parasites were thawed and maintained through serial blood passage in mice at the KEMRI animal facility, Nairobi, Kenya. Prior to resistance induction, susceptibility of the parental parasite line to AL was confirmed using the standard 4‐day suppressive test (4‐DT), followed by monitoring parasite progression for 15 days′ postinfection (dpi). Parasitized red blood cells (PRBCs) were harvested from donor mice exhibiting parasitaemia levels of 5%–10%. Based on parasitaemia counts, blood samples were diluted in phosphate saline glucose (PSG) buffer to prepare an inoculum containing approximately 2 × 10^7^ PRBCs in 0.2 mL.

### 2.2. Drug Preparation

Experimental ART and LM powder (≥ 98% purity; Sigma‐Aldrich, United States) and AL powder (Cofanart, Batch No. D25L005, Zain Pharma Ltd, Kenya) were used in this study. The reconstituted AL suspension contained 15‐mg ART and 90‐mg LM per 5 mL, corresponding to the standard 1:6 ART:LM ratio. On the day of administration, ART, LM and AL were freshly prepared by dissolving them in a solvent mixture containing 70% Tween‐80 (density = 1.08 g/mL) and 30% ethanol (density = 0.81 g/mL). The resulting solution was then diluted 10‐fold with double‐distilled to prepare the required working concentrations (mg/kg body weight) for each experimental dose immediately before oral administration.

### 2.3. Preparation of Inoculum and Infection Procedures

Parasite identity and purity were confirmed microscopically using Giemsa‐stained thin blood smears and further verified by species‐specific PCR amplification. Donor mice with parasitemia levels of 5%–10% were used for inoculum preparation. Blood collected from the donor mice was diluted with PSG buffer to achieve approximately 2 × 10^7^ PRBCs per 0.2 mL inoculum. During the experiment, mice were randomly assigned to the uninfected control, infected untreated control, and infected treated groups using a simple randomization procedure. Allocation was performed by an investigator who was not involved in subsequent data collection. Each mouse in the infected groups was inoculated intraperitoneally with 0.2 mL of infected blood containing approximately 1 × 10^7^ s PRBCs [26]. Where applicable, investigators assessing parasitaemia and treatment outcomes were blinded to group allocation. The study was conducted and reported in accordance with the ARRIVE 2.0 guidelines [27].

### 2.4. Determination of Effective Dose Levels (ED_50_ and ED_90_)

The effective doses required to reduce parasitaemia by 50% (ED_50_) and 90% (ED_90_) were determined using the standard 4‐DT [28]. Male Swiss albino mice weighing 18–22 g were randomly assigned into four treatment groups consisting of five mice (*n* = 5) per dose level and one untreated control group of five mice (*n* = 5). Baseline ED_50_ and ED_90_ values were determined using groups of five mice (*n* = 5) to generate reliable dose–response estimates. On Day 0, each mouse was inoculated intraperitoneally with 2 × 10^7^ PRBCs suspended in 200 *μ*L inoculum. AL was administered orally at 4, 24, 48, and 72 h postinfection (pi).

On Day 4 pi, thin blood smears prepared from tail blood were fixed in methanol and stained with freshly prepared 10% Giemsa solution for 10 min. Parasitaemia was determined microscopically from stained blood films, and percentage chemosuppression was calculated as previously described [29]. Percentage chemosuppression [30] was calculated using the formula: *%*chemo − suppression = [(*A* − *B*)/*A*] × 100, where *A* is the mean parasitaemia of the untreated control group and *B* is the mean parasitaemia of the treated group. Parasite counts were processed using Microsoft Excel, whereas ED_50_ and ED_90_ values were estimated graphically through linear regression analysis using GraphPad Prism v.11.0.2 (GraphPad Software, United States).

### 2.5. In Vivo Selection of AL Resistant Parasites

The ED_90_ dose was selected as the initial drug pressure for induction of AL resistance in *P. berghei ANKA*. Following parasite revival and two stabilization passages, three male Swiss albino mice were inoculated intraperitoneally with infected erythrocytes. The experimentally determined ED_90_ dose of AL (2.80 mg/kg) was used to initiate in vivo resistance selection once the parasitaemia exceeded 2%. During serial passaging, groups of three mice (*n* = 3) were used to maintain parasite propagation under continuous drug pressure. Parasites recovered from mice with the highest parasitaemia were serially passed into naïve mice for subsequent selection cycles. Drug pressure was maintained for the first five passages at 2.80 mg/kg and thereafter gradually increased from 5 mg/kg to a maximum of 15 mg/kg depending on parasite survival and growth dynamics. At every passage, three mice were infected and monitored for parasitaemia before drug administration. The ED_50_ and ED_90_ values were evaluated after every five selection passages to assess changes in parasite susceptibility.

The antimalarial response of the selected 20th passage *P. berghei ANKA* line was evaluated using the standard 4‐DT [19] following continuous selection under AL pressure. Three experimental groups were included: untreated parent parasites (negative control), parent parasites treated with 5 mg/kg AL (positive control), and the 20th AL selected parasite line treated with 5 mg/kg AL. Mice were inoculated intraperitoneally with infected erythrocytes and treatment was initiated according to the standard 4‐day suppressive model [28]. Parasitaemia levels were monitored at Days 0, 4, 7, 9, 11, and 15 pi to assess parasite growth and responsiveness to drug treatment [31].

### 2.6. Stability Test of Resistant Parasite Lines

The stability of the resistant *P. berghei* lines was evaluated by assessing parasite responses after removal of drug pressure [21]. Stability was determined through five consecutive drug‐free passages in mice, followed by freeze–thaw recovery of parasites cryopreserved at −80°C for 1 month. After revival, the parasites were subjected to the standard 4‐DT to reassess drug sensitivity and resistance levels.

Stable resistance was defined as the maintenance of the resistant phenotype after withdrawal of drug selection pressure for at least five passages in mice. Resistance indices at 50% and 90% inhibitory levels (RI_50_ and RI_90_, hereafter resistance index at the 50% or 90% inhibitory level) were calculated as the ratio of ED_50_ or ED_90_ values of the resistant strain relative to the parental strain. Resistance classification followed previously established criteria, where I_90_ values of 1.0–10.0 indicated slight resistance, 10.01–100.0 moderate resistance, and values greater than 100 indicated high resistance [32]. For the purposes of this study, these thresholds were used as standardized phenotypic benchmarks for comparing shifts in antimalarial drug susceptibility across experimentally selected parasite lines and for quantifying the magnitude of resistance relative to the parental strain.

### 2.7. Data Analysis

Data were recorded in Microsoft Excel and analyzed graphically to estimate the ED_50_ and ED_90_ values using the linear regression method using GraphPad Prism v.11.0.2 (GraphPad Software, United States) [21]. Resistance was categorized into four groups according to the criteria formerly described [19]: RI90 = 1.0 was classified as sensitive, RI90 = 1.01–10.0 as slight resistance, RI90 = 10.01–100.0 as moderate resistance, and RI90 > 100.0 as high resistance [33, 34]. The ED_50_ and ED_90_ values of the drug‐sensitive parental line and the AL‐resistant line was compared using Student′s *t*‐test. Differences between the parental and resistant lines were compared using Student′s *t*‐test and considered statistically significant at *p* < 0.05.

## 3. Results

### 3.1. Acquisition of AL Resistance

Before exposure of the parasite to AL drug pressure, the effective doses that suppressed parasitaemia by 50% (ED_50_) and 90% (ED_90_) were determined using the standard 4‐DT. The ED_50_ and ED_90_ values of the parent *P. berghei* strain were found to be 1.45 and 2.80 mg/kg, respectively (Table 1, Figure 1).

**Table 1:** Response pattern of the *Plasmodium berghei ANKA* resistant clone under continuous AL drug pressure in mice.

Passage no.	ED_50_ (mg/kg)	RI_50_	ED_90_ (mg/kg)	RI_90_
Parent	1.45	1.00	2.80	1.00
5th	1.92	1.32	3.44	1.23
10th	2.59	1.79	4.63	1.65
15th	2.95	2.03	5.28	1.89
20th	4.13^a^	2.85	7.42^a^	2.65

*Note:* Table 1 describes the evolution of AL resistance in *P. berghei ANKA* strain using serial technique. Data were presented as effective doses that suppressed parasitaemia by 50% and 90% (ED_50_, ED_90_) and as 50% and 90% indices of resistance (RI_50_ and RI_90_, defined as the ratio of the ED_50_ or ED_90_ of the resistant line to that of the parent strain).

^a^Significant differences in ED_90_ between the parent (sensitive) and resistant (20th in vivo drug selection passage) lines were determined using Student′s*t*‐test.*p* < 0.05was considered statistically significant.

**Figure 1:**
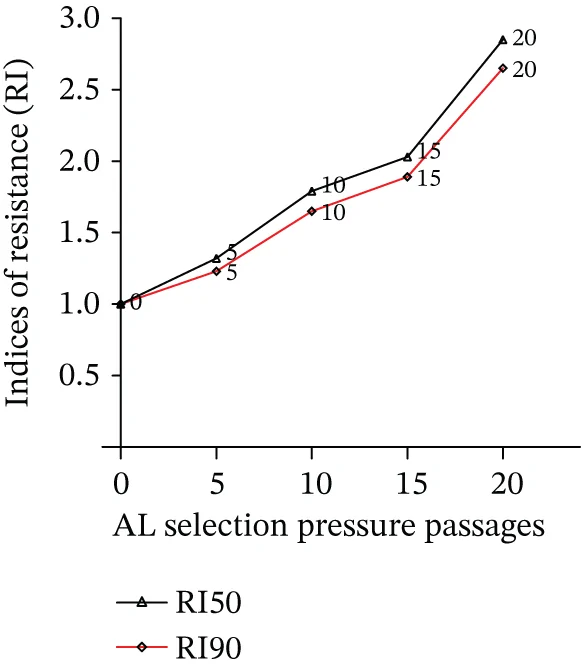
Graph showing increase in level of resistance. Level of resistance was assessed after every five (5) passages during the selection of AL resistance for over a period of 6 months. The index of resistance (I_50_ and I_90_) indicates the degree of parasites resistance to AL and is clearly the ratio of the ED_50_ or ED_90_ of the resistant line to that of the parent strain.

Following exposure to the initial AL selection pressure of 2.80 mg/kg, parasitaemia decreased from 6.20% at Day 5 pi to 2.1% 5 days after drug administration, indicating partial suppression of parasite growth. However, parasitaemia subsequently increased to 8.68% by Day 13 pi, suggesting survival and regrowth of parasite subpopulations under drug pressure. From the first and subsequent passages, the selection dose was gradually increased within a range of 2.80–15 mg/kg depending on parasite growth and survival. During the first five consecutive drug‐pressure passages, the average interval between subsequent passages was approximately 11–13 days. As selection progressed, this interval gradually reduced to an average of 8 days, indicating improved parasite adaptation and recovery under continued drug exposure.

Assessment of parasite susceptibility after every five‐selection passages revealed progressive alterations in responsiveness to AL (Figure 1; Table 1). At the early stage of selection (5th passage), only modest changes in susceptibility were observed, with ED_50_ increasing from 1.45 mg/kg in the parent strain to 1.92 mg/kg (RI_50_ = 1.32), whereas ED_90_ increased slightly to 3.44 mg/kg (RI_90_ = 1.23). By the 10th passage, a more pronounced reduction in susceptibility was evident, as reflected by increases in ED_50_ to 2.59 mg/kg (RI_50_ = 1.79) and ED_90_ to 4.63 mg/kg (RI_90_ = 1.65). This trend continued through the 15th passage, where ED_50_ and ED_90_ increased to 2.95 mg/kg (RI_50_ = 2.03) and 5.28 mg/kg (RI_90_ = 1.89), respectively, indicating gradual adaptation of the parasite population under sustained drug pressure. In summary, RI_50_ and RI_90_ increased progressively with successive AL selection passages, reaching 2.85‐fold and 2.65‐fold, respectively, at passage 20, indicating a progressive reduction in AL sensitivity (Figure 1).

By the 20th passage, the parasite line exhibited the greatest reduction in AL susceptibility observed in this study, with ED_50_ and ED_90_ increasing to 4.13 mg/kg (RI_50_ = 2.85) and 7.42 mg/kg (RI_90_ = 2.65), respectively. Although these findings demonstrate substantial reduction in drug sensitivity relative to the parent strain, they do not necessarily represent maximal resistance, but rather indicate progressive selection of parasite populations with reduced susceptibility under continuous AL pressure. The selected parasite population was designated as the 20th AL‐resistant (20th AL^R^) line.

To assess the response of the 20th AL‐resistant (20th AL^R^) *P. berghei ANKA* line to a high, well‐tolerated dose, the parasites were subjected to 5 mg/kg AL and compared against untreated parent parasites (negative control) and parent parasites treated with the same 5 mg/kg dose (positive control). Parasitaemia levels were monitored at Days 0, 4, 7, 9, 11, and 15 pi. In the untreated parent parasite group, parasitaemia increased progressively from 1.02% at Day 4 to 18.38% by Day 15 pi, indicating unrestricted parasite proliferation. In contrast, the AL treated parent parasites showed marked suppression of parasite growth, with parasitaemia declining from 2.65% at Day 7 to complete clearance by Day 11 pi (Figure 2). However, the 20th AL^R^ line continued to grow despite exposure to the 5 mg/kg dose, with parasitaemia increasing progressively from 0.94% at Day 4 to 10.8% by Day 15 pi.

**Figure 2:**
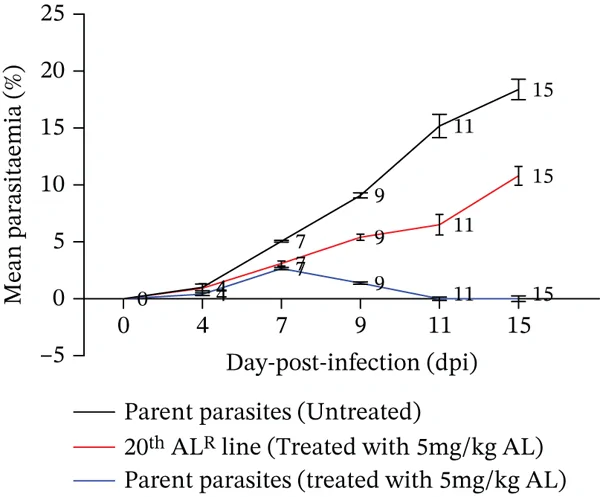
Growth profile of the 20th AL‐resistant (20th AL^R^) *Plasmodium berghei ANKA* line following exposure to a high‐dose AL (5 mg/kg) regimen during the standard 4‐day suppressive test. Parasitaemia progression was monitored on Days 0, 4, 7, 9, 11, and 15 postinfection and compared among the 20th AL^R^ parasite line treated with 5 mg/kg AL, untreated parent parasites (negative control), and parent parasites treated with 5 mg/kg AL (positive control). Error bars represent the standard error of the mean (SEM) across biological replicates.

In conclusion, Student′s *t*‐test comparison of the 20th‐passage AL‐resistant line and the parent sensitive parasites treated with 5 mg/kg AL showed significant differences in parasitaemia at Day 4 (*t*(8) = 3.278, *p* = 0.0112), Day 7 (*t*(8) = 4.755, *p* = 0.0014), Day 9 (*t*(8) = 37.289, *p* < 0.0001), Day 11 (*t*(8) = 19.391, *p* < 0.0001), and Day 15 (*t*(8) = 3.432, *p* < 0.0001). Thus, all pi time points showed statistically significant differences at *p* < 0.05, with the 20th‐passage resistant line generally showing substantially higher parasitaemia under 5 mg/kg AL treatment. This confirms that we successfully generated an AL resistant phenotype through continuous drug pressure over 20 serial passages. Progressive increases in ED_50_, ED_90_, and resistance indices indicate gradual parasite adaptation to AL exposure rather than the development of complete resistance. The 20th AL^R^ line retained the ability to proliferate under a high AL dose of 5 mg/kg, whereas the treated parental line was completely cleared. This differential response confirms reduced susceptibility of the 20th AL^R^ line to AL. However, the continued suppression of parasite growth indicates that the reduction in susceptibility remained slight and incomplete, consistent with the RI_90_ classification used in this study. These findings suggest an early‐stage adaptive phenotype arising under sustained AL selection pressure.

### 3.2. Stability Test

Following withdrawal of drug pressure, the resistant parasite line was subjected to five drug‐free passages in order to assess stability of the resistance phenotype. After drug‐free passages, the effective doses were determined to be ED_50_ = 3.98 mg/kg and ED_90_ = 5.58 mg/kg, corresponding to RI_50_ = 2.72 and RI_90_ = 2.35, respectively (Table 2). Although these values were slightly lower than those observed at the 20th passage under continuous drug pressure, they remained elevated relative to the parent strain. This indicated incomplete reversion to baseline sensitivity and suggested persistence of reduced susceptibility even in the absence of selective drug pressure. Besides, after 45 days of cryopreservation, the resistant parasite line retained reduced susceptibility to the drug, with effective doses of ED_50_ = 4.01 mg/kg and ED_90_ = 7.03 mg/kg, corresponding to RI_50_ = 2.77 and RI_90_ = 2.51, respectively. These values indicated that the resistance phenotype remained stable despite prolonged cryopreservation. Although slight variations in effective dose values may occur following storage and recovery, the resistance indices remained markedly elevated relative to the parent strain, demonstrating persistence of the reduced susceptibility phenotype after cryopreservation.

**Table 2:** Stability of AL‐resistance in *P. berghei* following drug‐free passages and cryopreservation recovery at −80°C.

Passage no.	ED_50_ (mg/kg)	RI_50_	ED_90_ (mg/kg)	RI_90_
Drug free passage	3.98	2.72	6.58	2.35
After 45days of cryopreservation	4.01	2.77	7.03	2.51

Despite the observed decrease in ED_50_ and ED_90_ after withdrawal of drug pressure and cryopreservation, the parasite line retained resistance characteristics, indicating partial stability of the AL resistance phenotype. The results suggest that reduced susceptibility to AL develops progressively under sustained drug pressure and may persist even after removal of the selective agent.

### 3.3. Reduced Sensitivity of AL‐Resistant Parasites to ART and LM Suggests a Combination‐Adapted Resistance Phenotype

To determine which component of AL was responsible for the observed resistance phenotype, four graded doses of ART and LM were independently evaluated against AL‐sensitive and AL‐resistant parasite lines using the 4‐DT. For ART, chemo‐suppression increased in a dose‐dependent manner in both parasite lines, but was consistently lower in the resistant line across all concentrations (Table 3). At 0.5, 1.0, 1.5, and 2.0 mg/kg, suppression in the sensitive parasites was 38%, 60%, 88%, and 96%, respectively, whereas the resistant line showed lower values of 32%, 40%, 48%, and 60% (Figure 3). This pattern indicates a progressive but incomplete loss of susceptibility to ART in the resistant parasites. A similar trend was observed for LM. At 1.5, 3.0, 4.5, and 6.0 mg/kg, chemo‐suppression in the sensitive line increased from 33% to 56%, 76%, and 90%, respectively (Figure 4). The dose ranges (0.5–2.0 mg/kg for ART, 1.5–6.0 mg/kg for LM, and 1.45–5.8 mg/kg for AL) were selected based on the experimentally determined ED_50_ values of the respective drugs.

**Table 3:** Chemo‐suppression of ART, LM, and AL in AL‐sensitive and AL‐resistant parasite lines.

ART			LM			AL		
Dose (mg/kg)	Sensitive	Resistant	Dose (mg/kg)	Sensitive	Resistant	Dose (mg/kg)	Sensitive	Resistant
0.5	38	32	1.5	33	20	1.45	36	16
1	60	40	3	56	34	2.9	56	20
1.5	88	48	4.5	76	42	4.35	87	31
2	96	60	6	90	48	5.8	100	34

*Note:* *n* = 5 mice per dose group.

**Figure 3:**
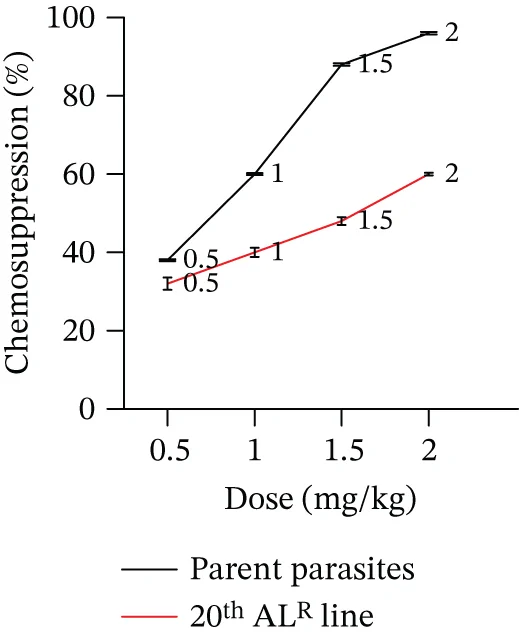
Chemo‐suppression of ART against parent parasites (sensitive) and AL‐resistant parasite line error bars represent the standard error of the mean (SEM) across biological replicates.

**Figure 4:**
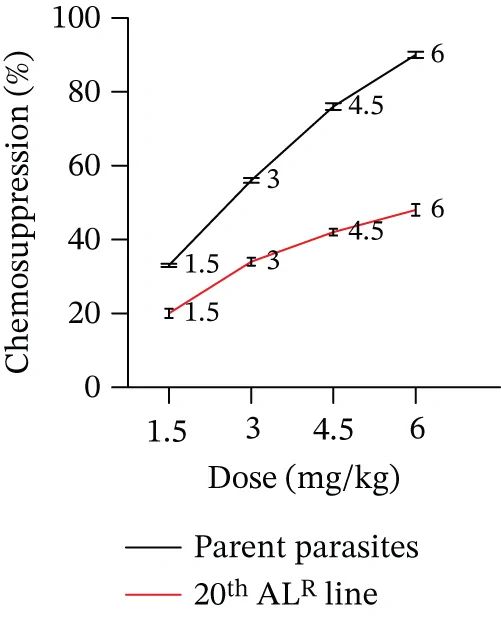
Chemo‐suppression of LM against parent parasites (sensitive) and AL‐resistant parasite line error bars represent the standard error of the mean (SEM) across biological replicates.

In contrast, the resistant parasites exhibited reduced suppression of 20%, 34%, 42%, and 48% at the same doses. Although LM retained partial activity, resistant parasites consistently survived at higher levels across all concentrations. When assessed as a fixed‐dose combination (Figure 5), AL demonstrated a markedly stronger suppression profile in the sensitive line (36%, 56%, 87%, and 100% at 1.45, 2.9, 4.35, and 5.8 mg/kg, respectively), whereas the resistant line showed substantially reduced suppression of 16%, 22%, 28%, and 34% (Figure 5).

**Figure 5:**
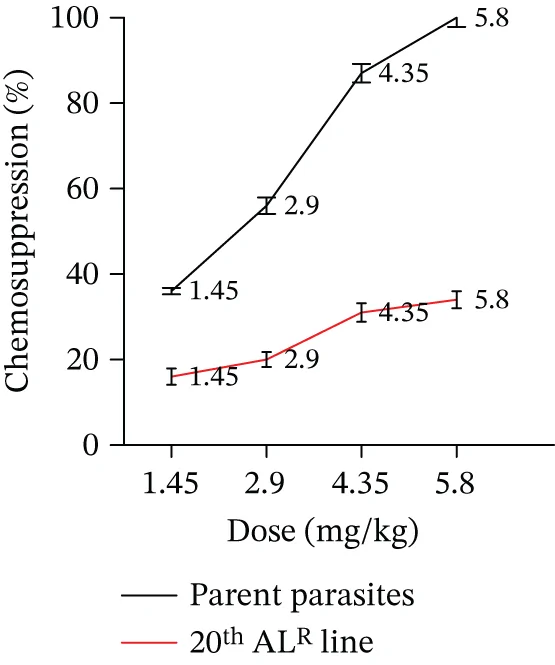
Chemo‐suppression of AL against parent parasites (sensitive) and AL‐resistant parasite lines error bars represent the standard error of the mean (SEM) across biological replicates.

Notably, the magnitude of the reduction in drug responsiveness was more pronounced against the combination than against either monotherapy individually. Overall, the resistant parasite line exhibited only partial reductions in susceptibility to ART and LM individually. However, the markedly diminished activity of the AL suggests a combination‐adapted resistance phenotype, where reduced drug sensitivity is amplified when both drugs are administered together. Based on the chemo‐suppression data, LM showed a proportionally greater reduction in resistant‐line responsiveness relative to the sensitive line than ART did at the corresponding highest dose tested (LM: 90%–48%, a 47% relative reduction; ART: 96%–60%, a 38% relative reduction).

## 4. Discussion

The present study demonstrated a progressive reduction in *P. berghei* susceptibility to AL following serial exposure to increasing drug concentrations. The ED_50_ and ED_90_ increased by 2.85‐fold and 2.65‐fold, respectively, following 20 passages under AL selection pressure, indicating a progressive reduction in parasite susceptibility. The reduced susceptibility was maintained following drug‐free passages and recovery after cryopreservation, although the phenotype showed some reduction compared with continuously selected parasites. These findings demonstrate that serial AL exposure selected parasite populations with reduced drug susceptibility under the conditions used in this study.

ACTs, especially artemisinin–lumefantrine (AL), are the main treatment for malaria, but reduced parasite susceptibility can still develop under prolonged drug pressure [35]. However, the process and early dynamics of resistance development under continuous AL exposure are not fully understood. This study is aimed at contributing to the understanding of how parasite susceptibility evolves under sustained AL selection pressure. The observed increase in ED_50_ from 1.45 mg/kg (parent) to 4.13 mg/kg (20th passage) and ED_90_ from 2.80 to 7.42 mg/kg indicates a stepwise selection of less drug‐sensitive parasite populations under sustained drug pressure. This gradual loss of susceptibility closely aligns with earlier experimental studies in *P. berghei*, including induction of LM‐ and piperaquine‐resistant [17] pyronaridine‐resistant [18–20] and ART‐resistant lines [21]. These studies demonstrated successful induction of resistance through serial drug exposure, with a generally progressive rather than abrupt emergence of reduced susceptibility. However, differences in the speed of resistance development have been reported, with some monotherapy‐induced resistance lines developing more rapidly than AL‐associated resistance, likely because of the stronger single‐drug selection pressure compared with combination regimens. In contrast, ACTs such as AL tend to slow resistance development because the two drugs exert different and complementary modes of action and pharmacokinetic profiles. Artemisinin acts rapidly with a short half‐life, whereas LM provides prolonged exposure, together reducing the probability of survival and slowing selection of fully resistant parasites [6].

Further, reported that LM‐ and PQ‐resistant *P. berghei* lines exhibit distinct fitness costs and stability patterns under drug‐free conditions [36]. Their findings showed that although PQ resistance can remain stable, LM‐associated resistance may be partially transient, highlighting variability in resistance persistence depending on drug pressure history and biological cost. More recently, Waithera et al. demonstrated that ART‐resistant *P. berghei ANKA* parasites can be successfully selected in vivo, but resistance development is associated with stepwise adaptation and measurable fitness trade‐offs [21]. In agreement with our study, after cryopreservation and 10 drug‐free passage the parasite lines showed partial reduction in susceptibility compared with continuously selected passages but did not fully revert to parent sensitivity, suggesting a measurable fitness cost and limited stability of the induced phenotype in the absence of drug pressure. Overall, this indicates that resistance is likely still at an early‐to‐intermediate stage of evolution rather than full clinical resistance saturation.

Our study demonstrated reduced responsiveness, rather than definitively established resistance, to both ART and LM, with latter showing a more pronounced reduction in efficacy at higher doses compared with former. Notably, the AL combination exhibited a disproportionately greater loss of activity against the resistant parasite line than either monotherapy, indicating an interaction‐level resistance phenotype rather than independent single‐drug resistance. These findings suggest that resistance is not driven solely by reduced sensitivity to individual components but likely involves coordinated mechanisms affecting both drugs, including altered hemoglobin digestion, enhanced cellular stress responses, and changes in drug transport or accumulation [37, 38]. The marked reduction in AL efficacy is particularly concerning given its role as a first‐line antimalarial therapy in endemic regions [39, 40]. Such combination‐adapted resistance may compromise treatment outcomes even when partial susceptibility to each drug persists. This observation aligns with reports of delayed parasite clearance and emerging ACT failures linked to reduced partner‐drug susceptibility in Southeast Asia and Africa [38, 41], underscoring the need for continuous surveillance. These findings are based on four graded dose levels tested for each drug, and individual ED_50_ values were not determined for ART and LM. Therefore, the observed effects are more appropriately described as reduced drug responsiveness under the experimental conditions rather than confirmed resistance.

Mechanistically, the observed phenotype reflects the contrasting pharmacological properties of ART and LM, which independently and jointly shape selection dynamics in ACT systems. ART is a short‐acting, fast‐killing endoperoxide with a very short half‐life (~1 h), producing rapid parasite clearance [42] through heme‐dependent activation and generation of reactive oxygen species, particularly targeting early ring stages. In contrast, LM is a long‐acting partner drug with a prolonged half‐life (3–6 days), acting mainly by inhibiting heme detoxification within the parasite digestive vacuole, leading to toxic heme accumulation [43]. This creates a temporal mismatch in drug exposure, where ART rapidly reduces parasite biomass, whereas LM maintains prolonged sublethal pressure on surviving parasites, favoring stepwise selection and survival of partially tolerant subpopulations.

As observed in our study, this dual pharmacological pressure may help explain the gradual increases in ED_50_ and ED_90_ observed across serial passages, consistent with cumulative accumulation of resistance traits over time rather than a single‐step genetic event. Consequently, we suggest that this progressive phenotype may be explained, at least in part, by classical resistance determinants such as *PfMDR1* amplification or polymorphisms, *PfCRT*‐associated vacuolar transport alterations, reduced hemoglobin digestion affecting ART activation, and enhanced oxidative stress tolerance mediated by antioxidant and heat‐shock responses [44]. These mechanistic explanations remain untested hypotheses rather than established conclusions in the present study. Emerging evidence from other systems also suggests a potential role for epigenetic regulation and transcriptional plasticity in early‐stage antimalarial tolerance, allowing parasites to transiently survive drug exposure before stable genetic fixation occurs [45, 46]. Such layered adaptation, if present, could plausibly help explain the gradual, nonlinear escalation in resistance indices and the partial persistence of reduced susceptibility even after drug‐free passage, although this remains an assumption requiring molecular validation.

In conclusion, this study provides evidence that serial AL exposure induces slight but progressive reductions in parasite susceptibility through multifactorial mechanisms involving drug transport, metabolic reprogramming, and stress‐response adaptation. However, the level of resistance achieved remains within the slight‐resistance category (per the RI_90_ classification used), indicating that fully stable high‐level resistance was not yet established. Therefore, this work lays a foundation for further investigations aimed at determining whether extended passage would lead to fixed resistance phenotypes. Future studies should focus on molecular characterization of key genes (*pfmdr1, pfcrt, kelch13*, and stress‐response regulators), assessment of resistance stability over extended drug‐free passages, and evaluation of potential cross‐resistance to other ACT partner drugs. Overall, although the findings are promising, they underscore the need for continued experimental and molecular surveillance of ACT resistance evolution.

We first acknowledge that *P. berghei* is a murine malaria parasite with important biological differences from *P. falciparum*; therefore, extrapolation of these findings to human malaria and clinical AL resistance should be made with caution. Second, no molecular or genomic validation, including sequencing of candidate resistance genes, gene expression analysis, copy number variation, or whole‐genome sequencing, was performed, limiting mechanistic interpretation and rendering the proposed mechanisms hypothesis generating rather than confirmatory. Third, the reduced‐susceptibility phenotype was achieved over a relatively limited number of selection passages and exhibited a modest resistance index, the long‐term stability and potential progression of which remain to be determined. Finally, parasite fitness was not assessed; therefore, any fitness costs associated with the reduced‐susceptibility phenotype remain unknown. These limitations provide important directions for future investigations.

## 5. Conclusions

Evolution of reduced AL susceptibility in *P. berghei* was successfully achieved through stepwise increases in drug concentration. We conclude that parasite lines with decreased susceptibility were selected; however, the reduction in susceptibility remained slight (per the RI_90_ classification) and not fully stabilized at high‐level resistance. AL‐resistant parasites provide a useful model for further molecular surveillance and mechanistic studies and may facilitate the development of strategies for monitoring and preventing the emergence of ACT resistance.

## Author Contributions

S.M.N., S.K.K., T.W.N., and M.S.O. conceived and conducted the experiment. M.W.W. performed data analysis and prepared draft manuscript. S.K.K. and T.W.N. provided supervision and funding support. J.N.W., J.S.W., and H.Y.W. provided expertise in drug action and development of resistance. All authors contributed to manuscript preparation and reviewed the final version.

## Funding

No specific funding was received for this research or the preparation of this manuscript.

## Disclosure

All authors approved the final draft and publication.

## Ethics Statement

All animal experiments were conducted in accordance with the ARRIVE guidelines and approved by the relevant Institutional Scientific and Ethics Review Committee (ISERC). The study was also authorized by the National Commission for Science, Technology and Innovation (NACOSTI; License No. NACOSTI/P/25/4182346).

## Conflicts of Interest

The authors declare no conflicts of interest.

## Data Availability

The data that support the findings of this study are available from the corresponding author upon reasonable request.
